# Digital Storytelling in Language Education

**DOI:** 10.3390/bs9120147

**Published:** 2019-12-09

**Authors:** Hamzeh Moradi, Hefang Chen

**Affiliations:** 1 College English Education Center, Nanfang College of Sun Yat-sen University, Guangzhou 51970, China; 2 College of Foreign Languages, Zhongkai University of Agriculture and Engineering, Guangzhou 51970, China; chenhefang@zhku.edu.cn

**Keywords:** digital storytelling, collaboration, communicative skills, language education, language enhancement

## Abstract

Modern technology provides lots of opportunities in order to connect classrooms with the world. Technology provides a greater and better source of information, yet solutions are needed to be mediated through the appropriate remedy. The emergence of new technology and digital resources during the past few decades has significantly influenced the learning environment and educational prospects. However, one of the challenges of practitioners and researchers is preparing learners with the required skills for the effective use of modern technology in the process of learning. Researchers proposed that a combination of societal constructivism and technology-integrated learning is crucial for obtaining and accomplishing present-day academic goals. The present paper highlights the significance and intricacy of modern technology, specifically digital storytelling (DST), in education. It elaborates the most salient aspects of DST application in language education, considering phases and elements of effective digital stories, steps of composing a digital story, and a critical description on the implementation of DST and fosterage of academic performance.

## 1. Introduction

Advances of modern technology in education, specifically in language education, result in a large amount of easy-access recourses, knowledge, and information to language learners [1] (p. 59). However, one of the most challenging facets of this technology adoption for the teachers, practitioners and researchers is preparing learners with the required skills for effective use of modern technologies in their process of learning. Researchers proposed that a combination of societal constructivism and technology-integrated learning is crucial for obtaining and accomplishing present-day academic goals [2,3]. The principles of social constructivist emphasize on the significance of learners’ effort and collaboration in utilizing the available learning activities, resources, and tools within the authentic and natural setting in constructing concepts, beliefs, and ideas [4]. Knowledge is not merely transmitted from teachers to learners; however, it is actually constructed by each and every student or group of students by their positive interaction with their social, physical, and technological environment [5]. Because modern technological devices are considered to be fundamental educational tools that have significant role in facilitating the construction of students’ knowledge, many researchers [6,7,8] have suggested the integration of information and communication technology (ICT) according to a theory called social constructivist.

Information technology integrated learning is a vital approach that can affect learning, teaching curricula, and materials [9]. However, sometimes, due to the insufficient knowledge and skills about ICT and modern technologies useful for pedagogy and education, ICT is applied incorrectly in the teaching process. For example, a study [10] focusing on the application of a multimedia storytelling website in foreign language learning in Taiwan depicts that, in addition to the problems related to curriculum, time limits, and workloads, language teachers in Taiwan have some additional difficulties, such as lacking or inadequate skills for the integration of stories into English language materials, finding and implanting suitable activities throughout the process of storytelling, and also a lack of training or experience in storytelling techniques.

A particular technology may have great pedagogical potential, but until it is applied properly, it may not positively affect the teaching-learning processes. Therefore, the effectiveness of technology for pedagogical purposes depends on the technology itself and on its users. Teachers may come across with different types of challenges for technology integration; examples can be their insufficient familiarity with modern technology, curriculum implementation problems, and lack of technological support [11].

Multimedia tools such as images, soundtracks, and video clips embedded into text or stories directly contribute and lead to the development of written digital stories [12]. Videos play a key role in DST, as they can be striking for both children and adults [13]. DST is a computer-based tool to tell a story [14].

The availability of low-cost, user-friendly, and advanced multimedia editing software and digital cameras (e.g., movie maker, Photo Story, and iMovie), as some of the technological achievements affecting education, suggests magnificent potential and capacity for creative teaching and learning. These kinds of multimedia presentation tools can be invaluable constructive means for transforming learners’ learning processes that focus mostly on production, collaboration, project management, group work, and critical thinking. Educators can provide extensive knowledge, which will provoke reflective thinking for successful transformative technology pedagogy and can also provide new ideas and alternatives for technology implementation and use [15].

Digital stories bring together graphics, sounds or recorded audio narration, music, and video to demonstrate information on a particular topic [16]. DST has a significant potential which may become an educational model for the present era [17].

Digital strolling (DST), benefiting from these kinds of breakthroughs in educational layout and technology, is becoming an encouraging support for a transformative technology approach for the improvement of learning, which includes content material, subject matter, critical thinking, information literacy, and motivation. Considering the fact that creating a promising digital storytelling (DST) project demands teachers to pose conditions that are significantly associated with contents of the course, learners are challenged with critical thinking about combinations of content material and multimedia components while considering the standpoint of audiences.

## 2. Methods

The paper foregrounds the significance and complexity of new technology, specifically DST, in language education. It aims at providing a systematic review for the use and implementation of digital storytelling in education, specifically language education; accordingly, it renders useful information and systematic procedures to design effective instructional activities based on teaching and learning objectives. The present review is limited to research published during 1978 to 2019, starting with [4] socio-constructive and socio-cultural theories in 1978, in which the principles of social constructivist, learning as socially interactive process and significance of learners were emphasized, to the most recent studies focusing on different facets of DST, from designing to integrating of it in education. With a consistent literature search using keywords, such as “digital storytelling,” “digital literacy,” “self-directed language learning (SDL),” “ICT in language education,” and “technology enhanced language learning,” 37 studies within this thematic index that explore the design, applications, and contributions of DST in different settings were finally identified to be reviewed in depth. Consequently, the paper depicts various phases of storytelling along with their sublevels, main elements for an effective digital story, steps for composing a digital story, and DST and academic achievement of the learners.

## 3. Results

### 3.1. Digital Storytelling

The implementation of educational technologies goes through a cultural shift among teachers who have responsibilities for the productive and effective implementation of technology in the teaching process [18]. “Preparing students to succeed in today’s increasingly global economy and complex world requires a shift from a teacher-centric culture to learner-centered instruction” [19].

Teachers also need to help students to perceive “contextualized differences in identity, culture, and language usage before practical integration of storytelling” [10]. Apart from social and cultural differences, for an efficient implementation of technology in education, the interaction between the quality of the technology nature itself, the content of curricular instruction, and the pedagogy used for conveying technology should all be carefully considered [20]. The implementation of new technology involves a number of stages, beginning with basic and fundamental awareness, and creates functional dimensions of administrative policy and maintenance, which finally leads to positive outcomes [21].

The advent of the internet and new technology has offered unheard-of potentials for classrooms connections, but the recent dispersion of digital cameras offers educational and instructional opportunities as well. Digital storytelling can be simply defined as telling stories in electronic form by combining text, audio, video, photos, music, etc. It is the process of writing about a story with the help of multimedia elements of music, voice imagery in order to create a visual story. Traditionally, storytelling was a powerful and significant means of education. Digital storytelling takes the ancient and traditional part of oral storytelling and involves a set of technical tools in order to create personal stories using graphics, sounds, music, and images together to accompany the authors’ voice [22].

As confirmed by the previous studies on DST, especially those reviewed in this paper, by integrating different types of multimedia, learners will be encouraged to tell even more fertile stories. By telling stories with the help of various types of new media, students learn how to deal with and manage information from various sources, and this enhances their communicative capabilities and information literacy. In addition, since in most cases digital storytelling can be done by learners in pairs or collaboratively in a small group, the students can strengthen and improve their collaboration and interpersonal communicative skills. By asking learners to gather information from various sources, instructors have the opportunity to get the students to explain and reflect the reasons why they made their selections, motivating them to become much more critically mindful about the process of learning and their particular choices. Digital storytelling is a precise activity that stimulates, encourages, and values activities that learners are engaging in outside of educational organizations and schools. It asserts that their skills are valuable and significant and can be used in schools in their process learning. The process of DST provides a high quality and significant learning experience. The implementation of technology in the teaching-learning process represents a significant approach where it extends and promotes the learning experience beyond what could be fulfilled and attained without technology. It enhances the multimedia and visual literacy of students, and it provides learners with a competitive compelling voice by elaborating the boundaries of learners who can communicate with and by enhancing the power and depth of that communication.

Many studies [3,23,24] have demonstrated that digital storytelling in fact goes far beyond the capability and functionality of traditional storytelling by generating and bringing in the learners’ concentrations, interests, and motivation, facilitating learner collaboration, group work, and organization of ideas, assisting learners to apprehend sophisticated learning material, and presenting information in a meaningful and versatile manner. DST helps learners to recapture, improve, intensify, apply, and extend creativity during the learning process. It assists learners in writing creatively and more effectively by visualization of their writing, which results in an additional level of perception and authentic personal learning that enhances the writing process and effective learning experience.

**Figure 1:**
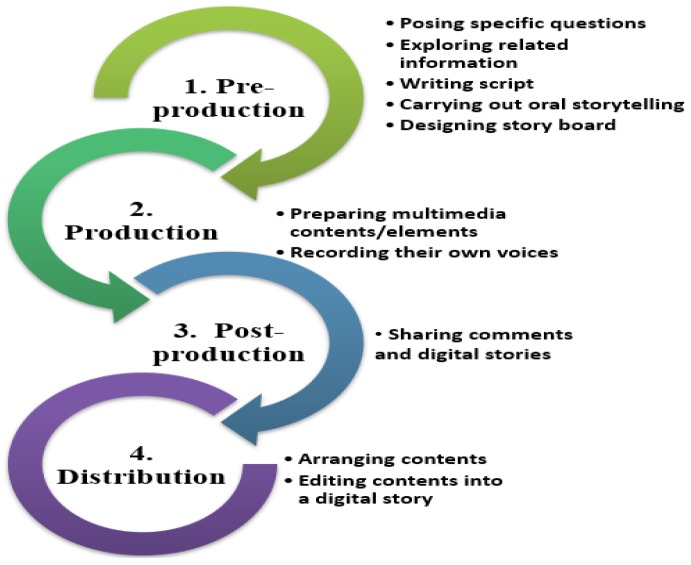
Phases of storytelling.

Digital storytelling provides a systematic procedure that helps educators to design effective instructional activities based on learning objectives. The following figure (Figure 1) illustrates four phases of storytelling along with the sublevels and activities of each phase.

To be in line with references [25,26], as shown in Figure 1, digital storytelling composed of four main phases, namely (a) preproduction (b) production (c) postproduction, and (d) distribution. Preproduction phase consists of five particular steps viz. posing questions, exploring topic-related information, writing script, and extracting peer review, presenting oral storytelling, and designing storyboard and story map. At the beginning, according to the context, background, experience, and interests of the students, the instructor tries to pose some particular questions in order to stimulate and motivate participants to pay attention to alternatives and make decisions upon a particular topic. Then, the learners can research appropriate information for the topic in order to write scripts that reflect and manifest the sequence of events. Next, the learners question each other and engage in peer review. Learners first attempt to practice saying their stories out loud in traditional ways that help in exploring the details of their stories. Then, a story map or storyboard is designed to demonstrate the story’s basic components.

The story map provides a straightforward and prompt assessment of learners’ stories and helps students to strengthen the weaker components of their stories. Furthermore, learners depict their own stories in a storyboard form, arranging the sequence of events, affect, scene and the rest of the digital elements. Throughout the production phase, learners create multimedia elements and also record their voices. Afterward, in the post-production phase, the content is arranged and also edited in a suitable manner in order to make a digital story. Then, in the distribution phase, the learners try to share their comments and produce digital stories with others. The dynamic and systematic procedure of creative storytelling encourages students to take a more active role in the process of learning and also enhances deeper connections with the learning materials and subject matter.

### 3.2. Elements of Effective Digital Stories

**Figure 2:**
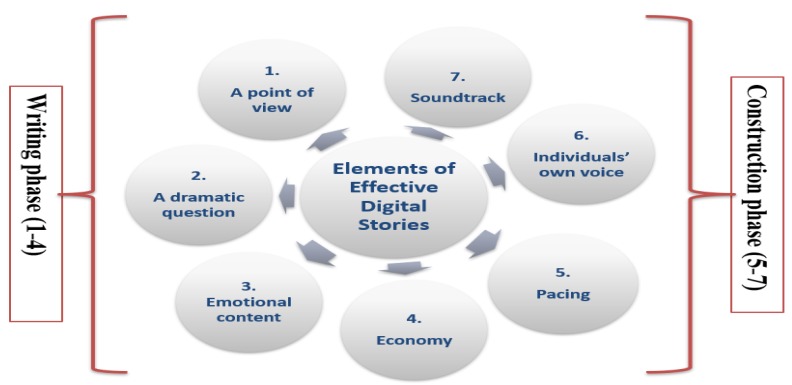
Elements of effective digital stories.

There are seven main elements for an effective digital story, including (1) a point of view, (2) a dramatic question, (3) emotional content, (4) economy, (5) pacing, (6) the gift of your voice, and finally (7) soundtrack. These elements can be classified in two main phases, namely the writing phase (1–4) and the construction phase (5–7), as illustrated in Figure 2, below.

During the planning and writing phase, learners attempt to make revisions in scripts and create storyboards [27]. They make decisions about the story content, what the story needs to say and how it will look like throughout this level. When the script and storyboard are ready, they make use of a digital video for the construction of the story. Each element of effective digital story is described below:

1. **Point of View:** Storytelling makes it possible for the writers to experience the possibility of using personal expressions. Thus, the digital stories of learners should be constructed from their personal experience and their own understanding. Instead of using the third-person pronoun, they should use the first-person pronoun or the first-person viewpoint for the construction of their digital stories. In other words, point of view demonstrates the perspective of the author and depicts the goal and the main point of the story.
2. **Dramatic Question:** A story that holds the audience’s attention poses a dramatic question which is answered or resolved by the end of the story. In other words, an effective digital story has a dramatic and key question that arouses the interest and concern of the audience and keeps the attention of the viewers.
3. **Emotional Content:** As the name suggests, emotional content, as one of the elements of effective digital storytelling, elicits emotions from the audience. When the stories are screened, we sometimes see the audience’s tears, laughter, and expressions of joy, which demonstrate the emotional content of effective digital storytelling and the connection of the story to the viewers.
4. **Economy:** Use only significant content and enough details to tell the story and not overload the audience with unnecessary information. Be precise, short, and simple in providing the content of the story. Economy is one of the most formidable elements of effective digital stories, where the writer needs to assiduously decide what is essential and crucial to the story.
5. **Pacing:** Pacing is related to the economy and particularly deals with how rapidly or slowly the story moves on and progresses. It concerns with the story’s rhythm; it should match with the purpose and objectives of the story.
6. **The Gift of Your Voice:** Students should try to personalize the story by recording themselves and narrating their own script in order to help the audience to perceive the content of the story.
7. **Soundtrack:** Incorporating music or other kinds of sounds supports and enhances the storyline and the depth of the narrative.

**Figure 3:**
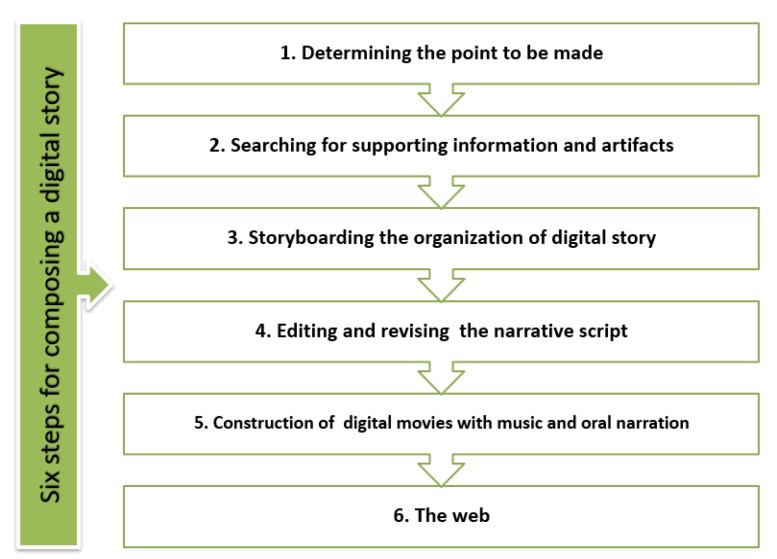
Steps of composing a digital story.

There are six steps for composing a digital story (as illustrated in Figure 3), including (1) determining the point to be made, (2) searching for supporting information and artifacts, (3) storyboarding the organization of the digital story, (4) group editing of the narrative script followed by revision, (5) construction of the digital movies with music and oral narration, and (6) the web [28].

In another study [29], it was revealed that students could make significant progress when their instruction moved from making a digital story to an individually improved personal narrative and a persuasive digital story. Throughout the creative story phase, the learners learned how to use the six steps of Kajder for composing a digital story, how to improve perspective, how to convert a written text into an oral narrative, and how to convert a storyboard into a digital movie [30].

1. **What to say:** Students need to up their minds about the point to be made and the story to be told. “They need to identify specific stories worth telling” [31].
2. **Artifact search:** Students need to search for the required and useful information and artifacts that can support and strengthen their stories.
3. **Storyboarding:** Learners need to storyboard the of digital story. This step has two dimensions—(1) chronologically (what happens and when) and (2) interaction (how audio information interacts with the presented images) [29]. The students need to map out each image, technique, and elements of their story by creating a storyboard. The storyboard also required the writer to consider how effects, transitions, and sound will be sequence [31].
4. **Revisions:** Students need to carefully examine their script to edit and revise the narrative, if required.
5. **Construction:** Students construct digital movies with music and oral narration. Students need to digitalize their photos, add particular effects, record narration, and add a soundtrack to make an effective digital story.
6. **Screening:** The final movie is published on the web or screened for an audience.

### 3.3. DST and Enhancement of Academic Achievement

During the last decade, the importance of digital storytelling in education and all levels of pedagogical practices have been drastically augmented. DST is a multidimensional learning approach for skills in critical thinking and reading, oral and written communication, and technology [30]. The application of DST in language education can improve language learners’ critical thinking, digital literacy, collaboration, their ability to make decisions, and their language learning skills [12,31]. Multiple kinds of literacy, such as digital literacy, visual literacy, technology literacy information literacy, and multicultural literacy, are accommodated and adapted by DST [23].

For the implementation of any kind of modern technology in education, a significant question that needs to be explored and should be kept in mind is how its implementation can affect the academic achievement of the learners; in other words, in order to use a new technology in teaching-learning process, instructors first need to assess and evaluate its impact on the academic achievement and performance of learners. With regard to language acquisition, researchers [32,33,34] assert that, at the early stage of language learning, academic achievement effectively correlates with the oral behaviors of singing, chanting, and repeating. Telling or even listening to stories can shape and influence early learning and the nature of intelligence [34]. Specifically, DST is considered to be effective in improving listening comprehension skills of the learners of English as a second/foreign language at the elementary level [35,36]. The authors of reference [16] assert that their findings confirm that DST can be very effective in enhancing writing skills of learners. They further state that the students’ commuter literacy and as well as their attitudes and motivations toward writing were significantly improved [17].

The integration of visual images with text can significantly improve learners’ comprehension, and DST is a particularly good technology tool to collect, create, analyze, and combining visual images with written text [36]. Therefore, DST as a relatively new technological tool in education can be used for developing and accelerating students’ comprehension capabilities. DST can facilitate the convergence of student-centered learning strategies, including student engagement, project-based learning, effective integration of technology into instruction, and reflection for deep learning [37]. Teachers are able to use DST to support the learning process of students by motivating them to organize and express their knowledge and point of view in an individually significant way. DST captures the imagination of students and as well as teachers and creates meaningful and significant digital stories can elevate and enhance experience for teachers and learners [24].

## 4. Discussion and Conclusions

The advent of new technologies and the implementation of them in education are significant challenges for educators and policy makers who need to enhance learning and teaching practices according to the needs of learners and academic objectives. Modern collaborative technologies make it possible for active production of shared knowledge.

Based on this review, it can be concluded that, with the help of modern technology, learners can actively become involved in their own learning process, the students’ cognitive development can be fostered, and collaborative learning and teaching practices can be enhanced significantly. Modern technology enhances self-directed learning (SDL). SDL gives language learners a greater role in their learning process [1].

To sum up, DST implementation in language education has many advantages, such as providing creative teaching methods, more variation than traditional approaches, personalizing learning experience, creating real-life situations in a simple and significant way, and engaging learners in the learning process. Integration of DST into the language curriculum is a significant step in language learning and teaching processes that can enhance students’ capacities for learning in the four skills of speaking, listening, reading, and writing. However, it should be mentioned that, besides all the positive findings regarding DST integration in education, more practical and extensive studies should be conducted in order to find out more about different aspects of DST. The next stage of our work will be a practical study on DST and its peculiar educational features and an experimental confirmation of this research.
